# Auxotrophic *Mycobacterium bovis* BCG: Updates and Perspectives

**DOI:** 10.3390/vaccines10050802

**Published:** 2022-05-19

**Authors:** Odir Antônio Dellagostin, Sibele Borsuk, Thaís Larré Oliveira, Fabiana Kömmling Seixas

**Affiliations:** Núcleo de Biotecnologia, Centro de Desenvolvimento Tecnológico, Universidade Federal de Pelotas, Pelotas 96010-610, RS, Brazil; sibele@ufpel.edu.br (S.B.); thais.larre@ufpel.edu.br (T.L.O.); fabianak@ufpel.edu.br (F.K.S.)

**Keywords:** auxotrophic BCG, recombinant BCG, live vaccine, tuberculosis, stability

## Abstract

*Mycobacterium bovis* BCG has been used for a century as the only licensed vaccine against tuberculosis. Owing to its strong adjuvant properties, BCG has also been employed as an oncological immunotherapeutic as well as a live vaccine vector against other pathogens. However, BCG vaccination has limited efficacy in protecting against adult forms of tuberculosis (TB), raises concerns about its safety in immunocompromised populations, compromises the diagnosis of TB through the tuberculin test and lacks predictability for successful antigen expression and immune responses to heterologous antigens. Together, these factors propelled the construction and evaluation of auxotrophic BCG strains. Auxotrophs of BCG have been developed from mutations in the genes required for their growth using different approaches and have shown the potential to provide a model to study *M. tuberculosis,* a more stable, safe, and effective alternative to BCG and a vector for the development of recombinant live vaccines, especially against HIV infection. In this review, we provide an overview of the strategies for developing and using the auxotrophic BCG strains in different scenarios.

## 1. Introduction

Bacille Calmette–Guérin (BCG) is a live strain of *Mycobacterium bovis* developed by Calmette and Guérin for use as an attenuated vaccine to prevent tuberculosis (TB) and other mycobacterial infections. The vaccine was first administered to humans in 1921 and remains the only vaccine against TB in general use. *Mycobacterium bovis* BCG is one of the most widely used vaccines worldwide and has already been administered to more than three billion people, especially in the setting of routine newborn immunization. This vaccine provides protection by inducing both humoral and cellular immune responses. Additionally, it has other desirable qualities for a vaccine; it is a potent adjuvant, safe, stable at room temperature, has a low production cost, can be administered soon after birth in a single dose, and also has the ability to induce nonspecific cross-protection against non-target pathogens by a mechanism known as trained immunity [1,2]. Advances in recombinant DNA technology, genomics, and the development of heterologous antigen expression systems in BCG make this bacterium a promising candidate as a multivalent recombinant vaccine vector expressing heterologous antigens from other microorganisms [3]. Moreover, considering BCG is a routinely administered vaccine, it constitutes a vector for the expression of heterologous antigens with the highest chances of being licensed for commercial use as a recombinant live vaccine.

However, BCG vaccination presents some issues related to its use, such as limited efficacy in protecting against adult forms of TB [4], concerns about safety for the immunocompromised population [5], and difficulties in TB diagnosis via the tuberculin skin test [6]. To overcome these issues, the attenuation of virulence has been explored by the development of auxotrophic BCG strains. In genetics, a strain is said to be auxotrophic if it carries a mutation that renders it unable to synthesize an essential compound required for its growth. Several auxotrophic mutants of BCG showed attenuation in comparison to the parental strain [7,8,9], which could be a safer alternative for the immunization of immunosuppressed populations, and did not sensitize a delayed-type hypersensitivity (DTH) response to the tuberculin test, that is, it did not preclude TB diagnosis [7].

**Figure 1:**
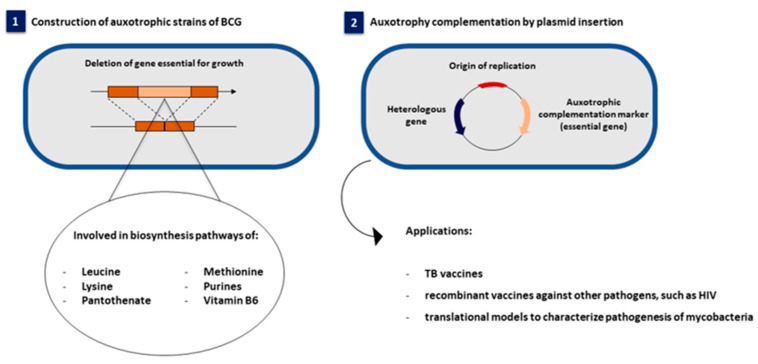
Schematic representation of the construction and applications of auxotrophic strains of BCG. (**1**) Development of auxotrophic strains of BCG from mutations or deletion of genes required for their growth, such as those involved in biosynthesis pathways of purines and amino acids; (**2**) Systems for auxotrophy complementation by plasmid insertion and applications of BCG auxotrophs.

Moreover, the use of antibiotic resistance markers in vectors used for the genetic transformation of BCG, which does not maintain selective pressure in vivo, limits vaccine stability and compromises the induction of long-lasting immune responses [10]. Alternatives to overcome antibiotic resistance have focused on the use of auxotrophic markers. Auxotrophic strains require external supplementation with essential compounds for their growth. The selection is performed using an expression vector containing the gene that complements the strain mutation (Figure 1) and restores the ability of BCG to grow in minimal medium without exogenous supplementation [11,12,13]. The use of auxotrophic complementation as a selectable marker generally provides higher stability rates in vivo, as the selective pressure for the vector is maintained and eliminates the problem of having an antibiotic resistance gene as part of the vector.

Several *M. bovis* BCG auxotrophic strains have been developed (Table 1). Most of them have disrupted genes required for the synthesis of essential amino acids, such as lysine, leucine, and methionine, or other components, such as purines, vitamin B6, and pantothenate, in the *M. bovis* genome. Auxotrophic mutants have been constructed using mycobacterial suicide plasmids for unmarked deletion [14], homologous recombination using mycobacteriophages [12,15], or transposon insertions [16]. In this review, we aim to provide an overview of strategies for the construction and use of auxotrophic BCG strains in different scenarios.

## 2. Targets for Construction and Applications of Auxotrophic BCG Strains

### 2.1. Lysine

L-lysine is an essential amino acid for human and animal nutrition. The *lysA* locus encodes the last enzyme involved in the lysine biosynthesis pathway. Because the expression of *lysA* depends only on the intracellular concentrations of lysine and diaminopimelate, the growth response of a mutant lacking *lysA* should be specific for extracellular lysine supplementation. Therefore, the deletion of the *lysA* gene leads to the loss of lysine synthesis, resulting in an auxotrophic strain for lysine [35].

A previous study described mutants constructed with deletions in the *lysA* gene using a mycobacterial suicidal plasmid bearing the counter-selectable marker *sacB* (pYUB657) for the allelic exchange of unmarked deletion mutations in two steps in the chromosomes of the substrains of *M. bovis* BCG and *M. tuberculosis* H37Rv, resulting in a lysine auxotroph of BCG [14]. This mutant was used to express the coding sequence of the HIVA gene (derived from the consensus HIV-1 clade A Gag protein), cloned into the pJH222 *E. coli*-mycobacterial shuttle plasmid that employs an antibiotic-free selection system, whose stability in vivo was demonstrated for at least 20 weeks [11]. This strain, expressing HIVA Gag p24/p17 domains and an immunodominant epitope from Env protein, under the control of the *Mtb* α-antigen promoter, was used for the prime vaccination of mice, followed by an MVA.HIVA boost. The vaccination regimen elicited HIV-1-specific CD8+ and tuberculin-specific T-cell responses in both adult and newborn mice, demonstrating its safety profile for the development of a dual pediatric vaccine platform against HIV-1 and TB [11]. Another study also reported the use of pJH222 episomal vector and the pJH223 integrative vector for the expression of the HIV-1 clade A-derived immunogen HIVA in a lysine auxotroph of BCG [23]. Higher levels of antigen expression were observed using the episomal vector pJH222.HIVA, and this construct was further evaluated in a priming-boosting regimen with heterologous components in mice. The rBCG vaccine elicited HIV-1-specific responses and protection against a surrogate virus challenge, as well as against an aerosol challenge with *M. tuberculosis*, characterized by a decrease in the bacterial load in the lungs and spleen [23]. 

Another study used the pJH222 plasmid to express HIV-1 genes. Two candidates were developed; in the first vaccine BCG.HIVA^401^, the *HIVA* gene was expressed in theDanish strain BCG_1331_Δ*ureC::ΩpfoA(G137Q)*, which expresses mutated detoxified perfringolysin O and facilitates BCG escape from the endosome and thus increases the availability of the transgenic protein for antigen processing and presentation. The *HIVA* gene was integrated into the mycobacterial chromosome and its transcription was driven by the *hsp60* promoter. The second vaccine, BCG.HIVA^222^, was derived from a lysine auxotroph strain of BCG Pasteur mc^2^1604Δ*lysA5::res*, and the gene encoding the enzyme required for the last step in lysine biosynthesis was supplied from the transformed episomal plasmid pJH222.HIVA [21]. BCG.HIVA^222^ primed HIV-1-specific CD8+ T-cell responses, enhanced by heterologous boosts with human adenovirus-vectored HAdV5.HIVA, sheep atadenovirus-vectored OAdV7.HIVA, or poxvirus MVA.HIVA vaccines. When the nonauxotrophic strain, BCG.HIVA^401^, harboring an integrative vector, was used, similar immunological properties were observed; however, vaccines elicited distinct profiles of intercellular signaling molecules produced by the immune splenocytes after HIV-1- and BCG-specific stimuli [21]. 

Other studies used the p2auxo.^int^ plasmid, a double auxotrophic *E. coli*–mycobacterial shuttle integration vector, which contains the *glyA* and *lysA* genes and functions as an antibiotic-free selection system in the auxotrophic strains of *E. coli* M15Δ*glyA* and BCG Δ*lys*, respectively. This vector was used to clone synthetic sequences of HIVconsv1 and HIVconsv2 fused to the 19-kDa lipoprotein secretion signal sequence, generating p2auxo.HIVconsv1^int^ and p2auxo.HIVconsv2^int^ plasmids containing sites for integration into the BCG genome [17]. The recombinant plasmids were transformed into BCG∆*lysA* and vaccine strains showed stability for 35 bacterial generations in vitro and for 7 weeks post-inoculation in vivo. Mahant et al. (2017) used the same integrative *E. coli-*mycobacterial shuttle plasmid p2auxo.^int^ to express the HIV-1 clade A immunogen HIVA, generating the BCG.HIVA2auxo.^int^ vaccine [18]. Saubi et al. (2014) engineered the mycobacterial shuttle plasmid p2auxo.HIVA for episomal replication, expressing the HIV-1 clade A immunogen HIVA in an auxotroph of the *M. bovis* BCG strain to generate the BCG.HIVA^2auxo^ vaccine and evaluated the BCG.HIVA^2auxo^ prime and modified vaccinia virus Ankara (MVA).HIVA boost [20]. The rBCG strain harboring p2auxo.HIVA^int^ was 8-fold more stable in vitro than the episomal plasmid. Moreover, the BCG.HIVA2auxo.^int^ vaccine, in combination with MVA, was safe and able to induce HIV-1 and *Mtb*-specific INF-γ-producing T-cell responses in adult mice [18]. In addition to the expression system used, Joseph et al. (2010) demonstrated the role of using weak promoters to control HIV-1gp120 gene expression and to provide auxotrophic complementation by plasmid insertion to prevent instability caused by genetic rearrangements in rBCG-based vaccines [22].

Approaches to prime vaccination with recombinant lysine BCG auxotrophs and boost with chimpanzee adenovirus (ChAdOx1) have also been explored, demonstrating the safety and induction of HIV-1-specific T-cell responses. The magnitude of total T-cell response [19] and of total frequencies of CD8+ T cells producing cytokines [17] were similar between animals primed with the rBCG or only with the parental BCG strain, probably due to the strong adjuvant properties of BCG and its ability to induce trained immunity. 

Auxotrophic BCG has also been used for the delivery of antigens from *Bordetella pertussis* [12]. A lysine auxotroph of BCG was obtained by disruption of the *lysA* gene by homologous recombination using mycobacteriophages to construct an unmarked complemented strain [12]. The complementation vector pNL71S1-*lysA*(kan^−^) was derived from pNL71S1PT [36] and comprised the upregulated *M. fortuitum* β-lactamase promoter and signal sequence, fused to the S1 subunit of the genetically detoxified *B. pertussis* toxin, PT-9K/129G. The BCG-Δ*lysA*-S1PT-LysA+ strain protected neonatal mice after an intracerebral challenge with *B. pertussis* by inducing a Th1-predominant immune response [12].

### 2.2. Leucine

L-Leucine is an essential branched-chain amino acid in humans and animals. L-leucine is synthesized from L-pyruvate, where seven enzymes are involved in the biosynthesis of L-leucine. The *leuABCD* cluster is composed of four genes that specify the structure of the three enzymes which constitute the *leu* operon in *E. coli.* The *leudC* and *leudD* genes encode the large and small subunits of 3-isopropylmalate dehydratase [37] and are the main targets for deletion in the genome of *M. bovis* BCG to generate auxotrophic mutants.

Borsuk et al. (2007) generated an unmarked *M. bovis* BCG Δ*leuD* and *M. smegmatis* Δ*leuD* by homologous recombination using the strategy of Parish and Stoker (2000), who developed a flexible cloning strategy based on two different vector series to separate the cloning (pNIL vector) and manipulation of the target gene from the inclusion of the required marker genes (pGOAL suicide vector) [38]. The same group described the construction of a BCG expression system using auxotrophic complementation with *leuD* as a selectable marker, resulting in the episomal plasmid pUP402 expressing antigens in *M. bovis* BCG Δ*leuD* [13]. The use of auxotrophic complementation as selective marker in an antibiotic resistance-*free* mechanism can improve the stability and safety of recombinant strains expressing heterologous antigens. The use of antibiotic resistance markers is an important tool for the selection of recombinant clones in vitro. However, for recombinant live vaccines, selective pressure is lost in vivo, which can compromise their genetic and functional stability and then the induction of long-lasting immune responses [10]. The rBCG strains carrying vectors containing auxotrophic complementation showed a 90–100% stability in mice over 30 weeks, compared to less than 20% observed after 5 weeks of inoculation when the kanamycin resistance gene was maintained [13].

The system described by Borsuk et al. (2007) was further used for the expression of antigens from *Leptospira interrogans* [24] and *M. bovis* [25]. Seixas et al. (2010) constructed two recombinant BCG Δ*leuD* strains, each expressing different leptospiral antigens. Both strains were 100% stable in mice after 18 weeks of the experiment, whereas the BCG Pasteur strains harboring the same plasmids showed only a 7% stability in vivo in the same period post-inoculation [24]. A recombinant BCG strain overexpressing Ag85B was also developed using the Δ*leuD* auxotrophic system and was evaluated as a vaccine against bovine tuberculosis. The vaccination of cattle with Δ*leuD* BCG-85B showed a significant reduction in histological damage to the lungs, as well as a stronger activation of CD4+ and CD8+ T-cell responses in comparison with the BCG Pasteur vaccination [25]. Moreover, the Δ*leuD* BCG-85B strain demonstrated a 94.24% rate of stability over 18 weeks post-inoculation, whereas only a 46.8% stability was observed for the nonauxotrophic strain, BCG Pasteur [26]. The BCG Δ*leuD* strain superexpressing Ag85B was also able to significantly inhibit the proliferation of the human bladder carcinoma cell line 5637 in comparison to the control strains (BCG Pasteur and BCG Δ*leuD* without plasmid) through the activation of proapoptotic and cell cycle-related genes, suggesting its potential as an immunotherapeutic agent for bladder cancer [27].

A previous study described the generation of two auxotrophs for leucine and one for methionine, which were isolated from a library of transposon insertions in BCG using IS1096-derived transposons. The transposon in the methionine auxotroph was inserted into an ORF with high homology to a family of sulfate-binding proteins, whereas in the two auxotrophs for leucine, sequence analysis and homology searches indicated that this gene encodes the BCG homolog of the product of the *leuD* gene in *E. coli*. When inoculated into C57BL/6 mice, the BCG leucine auxotrophs were unable to grow in vivo, in contrast to the parental BCG strain or the methionine auxotroph. The complementation of the leucine auxotrophs was performed using the *leuCD* from *E. coli* by cloning into the mycobacterial expression vectors pMV261 and pMV361, generating pYUB425 [16]. A subsequent study demonstrated that this mutant BCG leucine auxotroph is incapable of replicating within cultured macrophages in vitro. Complementation of the *leuD* mutation restored wild-type growth rate in macrophages, showing that the defect for intracellular replication was due to leucine auxotrophy per se and not due to a polar effect of the transposon insertion on an adjacent gene [28]. The authors suggest using this in vitro platform for the screening of auxotrophic strains of *M. bovis* BCG or *M. tuberculosis* as potential new vaccines against tuberculosis before animal experiments [28].

The reduced ability of BCG leucine auxotrophic strains to establish infection in mice led Grandoni et al. (1998) to hypothesize that inhibitors of branched-chain amino acid biosynthesis could be repositioned for the treatment of *M. tuberculosis* infections. Herbicides that inhibit acetolactate synthase (ALS) and ketol acid reducto isomerase (KARI), branched-chain amino acid biosynthetic enzymes, were evaluated for the growth inhibition of an *M. tuberculosis* ATCC strain as well as clinical isolates. Sensitivity to compounds was demonstrated only for clinically drug-resistant isolates, with no significant effectiveness in inhibiting the growth of the ATCC strain. These studies highlight that the biology and physiology of BCG auxotrophs can be explored as models to characterize the pathogenesis and virulence of mycobacteria [28], as well as to evaluate potential antituberculosis drugs [39].

In another study, guinea pigs vaccinated with the leucine auxotroph of BCG were protected against infection from *M. bovis,* in terms of number and size of pulmonary lesions and the presence of mycobacteria in spleen, while animals infected with *M. tuberculosis* were protected only against hematogenous spread. In addition to protection, no induration after the tuberculin skin test was observed in animals inoculated with the leucine auxotroph, which was related to its low persistence in vivo. These findings highlight the attenuation of the leucine auxotrophic mutant as a way to develop safer TB vaccines for immunosuppressed, and the absence of sensitization to tuberculin as a possible way to investigate mechanisms involved in vaccine protective immunity and in DTH cutaneous reaction, without precluding the use of the tuberculin skin test for diagnosis and control of TB [7]. 

Specialized phage transduction unmarked using a conditionally replicating mycobacteriophage containing a γδ resolvase gene, was used to generate the auxotrophic strains of *M. bovis* BCG Danish (BCGD) Δ*leuCD* [34]. This mutant was used to stably express the lentiviral antigens, human immunodeficiency virus (HIV) gp120, and simian immunodeficiency virus (SIV) Gag, using selectable leucine auxotrophic complementation using the pYUB2115 plasmid [29]. Considering the wide range of studies using auxotrophic strains of BCG as vaccine vectors against HIV, as reviewed here, and the need for reproducibility and predictability for their use as vaccine candidates, Hart et al. (2015) established a vaccine production protocol and quality control parameters applied to stable lentiviral antigen expression in a leucine auxotrophic BCG strain [29]. These parameters, including the monitoring of optical density, clumping assessment by microscopy, purity determination by streaking the lot material on chocolate agar, the detection of antigen expression, and in vitro stability by western blotting and analysis of plasmid retention, were predictors of stability and successful antigen expression in vivo as well as immune responses induced in mice.

### 2.3. Pantothenate

Pantothenate is a key precursor of coenzyme A and an acyl carrier protein which is essential for many intracellular processes, including fatty acid metabolism, cell signaling, and synthesis of polyketides and non-ribosomal peptides [40]. The products of genes *panD* (aspartate-1-decarboxylase), *panB* (ketopantoate hydroxymethyltransferase), *panE* (ketopantoate reductase) and *panC* (pantothenate synthetase) are required for the formation of pantothenate [40]. The auxotroph BCG strains for pantothenate have disruption of genes *panD* and *panC,* which play a role in the first and last steps of the biosynthesis of pantothenic acid.

A study by Sambandamurthy et al. (2002) described an attenuated pantothenate auxotrophic strain (BCGΔ*panCD*), a mutant for *panC* and *panD* genes [41]. For this, an 823-bp region upstream of the *panC* gene and a 716-bp region downstream of the *panD* gene were cloned into pJSC347, flanking a hygromycin cassette to create pSKV1. *Pac*I-digested pSKV1 was ligated into temperature-sensitive mycobacteriophage phAE159 derived from TM4 and transduced to generate auxotrophs. To complement the mutation, the *panCD* operon was subcloned into pMV306kan, an integrating mycobacterial vector [41]. This attenuated pantothenate auxotroph strain (BCGΔ*panCD*) has been used in some studies to express HIV genes, aiming to develop safer combined TB-HIV vaccines for immunocompromised, elderly, and infant populations [30,31,32]. 

Recombinant pantothenate auxotroph strains of BCG expressing Gag, RT, or truncated Env (Gp120) proteins were used for the prime vaccination of mice, followed by boosting with the modified vaccinia virus Ankara (MVA) expressing the same antigens [30]. When administered individually or in a mixed formulation, rBCG auxotrophic strains primed the immune system and induced HIV-specific T cell responses. Moreover, all rBCG vaccines were stable in vivo and in vitro, despite the plasmids not complementing the auxotrophic mutation and using only kanamycin resistance as a selective marker. A previous study reported the instability of this auxotroph, characterized by plasmid deletions and rearrangements, when the plasmid pHS300, containing the *lysA* gene under control of *hsp60* promoter, was used. Instability is associated with the activity of the strong *hsp60* promoter, which has already been reported in other studies. The use of a codon-optimized *gag* gene and deletion of the *hsp60* promoter and *lysA* gene from the vector, keeping only the antibiotic resistance marker, improved vaccine stability [32], which was also able to elicit specific CD8+ and CD4+ T cell responses that protected against vaccinia virus surrogate challenge. 

The recombinant pantothenate auxotroph expressing (HIV-1) subtype C Gag protein was also evaluated in a prime immunization in nonhuman primates boosted with HIV-1 Pr55^gag^ virus-like particles (Gag VLPs) [31]. The heterologous prime–boost rBCGΔ*panCD*-Gag-VLP vaccine scheme was highly immunogenic and able to elicit Gag-specific polyfunctional and central memory T cell responses, characterized by the production of IFN-γ, TNF-α, and IL-2, as well as to induce HIV-1 antibody responses. When rBCGΔ*panCD*-Gag and MVA-Gag were used in a heterologous prime–boost regimen, these cytokines were also produced at higher levels than those observed in mice that received a homologous vaccination scheme or prime immunization with BCG parental; however, only effector memory T cell responses were observed [33]. This confirmed the ability of rBCGΔ*panCD*-Gag auxotrophs to efficiently prime the immune system for a heterologous boost with MVA-Gag.

### 2.4. Methionine

Methionine biosynthesis comprises two biosynthetic domains: (i) sulfur assimilation, reduction, and cysteine biosynthesis and (ii) one branch of the biosynthetic pathway of the aspartate amino acid family [42]. The *cysA*^−^ mutants are cysteine auxotrophs that indirectly affect methionine synthesis, justifying the description of mutants for methionine by disruption of the *cys* gene. A previous study described mutant generation using the mycobacteriophage phAE77 and an *E. coli* cosmid vector carrying Tn*5367* that had been inserted into a nonessential region of the D29 mycobacteriophage genome. A total of 1500 mutants from the arrayed BCG library were screened for auxotrophy by plating on media with or without added amino acids. This yielded two auxotrophs that required amino acid supplementation for growth. Their auxotrophy was confirmed by their failure to grow after restreaking on fresh minimal media. They were then subjected to auxanogram analysis, which revealed that both auxotrophs require methionine [15]. 

The use of these mutants allowed the characterization of sulfur metabolism in mycobacteria, demonstrating the application of BCG auxotrophs as a translational model for study of mycobacteria. The *cysA* mutant of *M. bovis* BCG was unable to take up sulfate in vitro and required methionine supplementation for growth, despite the presence of functional *cysA2* and *cysA3*. Methionine auxotrophy was confirmed when a plasmid containing the *cysA* gene was inserted into the mutant strain, and its ability to grow without amino acid supplements was restored, indicating that this is the single locus responsible for inorganic sulfur transport. Despite impaired growth in vitro, no attenuation was observed in the auxotrophic strain in vivo [15].

### 2.5. Purines

Purines have several important physiological functions as nucleic acids constituents and as intracellular and extracellular signaling molecules. Their biosynthesis requires ten enzymatic transformations to generate inosine monophosphate. PurF, PurD, PurL, PurM, PurC, and PurB were common to all pathways [43]. Only one study has described auxotrophic mutants of *M. tuberculosis* and *M. bovis* BCG by the disruption of one of their purine biosynthetic genes. The plasmid pMJ111 was created by excising the 1,090-bp *Eco*RV-*Hin*cII fragment from the pMJ1 plasmid, harboring the *purC* gene flanked by 120 bp upstream and 80 bp downstream, and cloning it into plasmid pOMK [44], resulting in a suicide vector (pMJ111) with a disrupted copy of the *purC* gene (*purC*::Km). The results showed that the mutants were unable to multiply in vitro in mouse bone marrow-derived macrophages compared with its parental strain and their growth in vitro was dependent on supplementation with purines or hypoxanthine [8]. In vivo, the purine auxotroph mutant showed the attenuation of virulence and the induction of a delayed-type hypersensitivity (DTH) response in the tuberculin test. *M. bovis* BCG *purC* mutant-vaccinated animals showed reduced mycobacterial burden in the lungs, but not in the spleen, after an aerosol challenge with *M. tuberculosis* H37Rv, suggesting the potential of auxotrophic BCG strains as safer vaccine candidates against tuberculosis, especially in vulnerable and immunocompromised individuals, such as infants and HIV-positive populations [8].

### 2.6. Vitamin B6

Vitamin B6 is an essential water-soluble cofactor in humans. In contrast, plants, fungi, and bacteria, including *M. tuberculosis*, synthesize pyridoxal-5-phosphate (PLP), the bioactive form of vitamin B6, from glutamine and derivatives of carbohydrate metabolism, in a two-step reaction catalyzed by Pdx1 (Rv2606c) and Pdx2 (Rv2604c). Whole-genome analysis predicted the ability of *M. tuberculosis* to synthesize vitamin B6, an essential component of the human diet [45]. 

A study reported that vitamin B6 biosynthesis is required for the survival of *M. tuberculosis* in vivo and thus might represent a candidate pathway for the development of new antitubercular agents. The deletion of the *pdx1* gene renders *M. tuberculosis* auxotrophic for vitamin B6 and markedly compromises its persistence in mice [46]. The *pdx1* gene was disrupted in a BCG strain previously engineered to secrete pore-forming listeriolysin O (BCG Δ*ureC*::*hly*). Fragments flanking *pdx1* were inserted into pYUB854, which was used for knocking out this gene in BCG Δ*ureC*::*hly* by electroporation. For genetic complementation, *pdx1*, including its putative promoter, was inserted into the integration vector pMV306. 

The knockout of *pdx1* promoted the attenuation of the auxotrophic strain in both immunocompetent and immunosuppressed mice when compared to the parental strain. Dietary supplementation with vitamin B6 partially restored the persistence of BCG Δ*ureC*::*hly* Δ*pdx1* in vivo, enhancing the induction of acquired immunity characterized by the generation of antigen-specific CD4 T cells and B cells, at levels equivalent to those observed in mice inoculated with BCG Δ*ureC*::*hly.* In mice vaccinated with a homologous prime–boost regimen and challenged with *M. tuberculosis*, BCG Δ*ureC*::*hly* Δ*pdx1* provided early reduction of bacterial burden in the lungs (30 days post-challenge) when a vitamin B6-enriched diet was supplied, suggesting the modulation of vaccine efficacy by pyridoxine dietary intake [9]. 

## 3. Perspectives

Auxotrophic BCG strains have been developed by mutating genes required for the synthesis of compounds essential for their growth. Such mutants have been widely used as models to study tuberculosis pathogenesis, develop safer TB vaccines, and express antigens from other microorganisms. Vaccine vectors based on auxotrophic strains may be safe and stable, be highly necessary for the induction of long-lasting immune responses, employ approaches such as codon-optimization, utilize promoters upregulated inside macrophages, employ signal sequences that drive antigen expression on the surface of mycobacteria, and utilize expression systems based on antibiotic-free selection. The attenuation and low persistence of auxotrophic strains in vivo also contribute to their safety and, in turn, their use in immunocompromised patients, while their immunogenicity can be balanced and strengthened through heterologous prime–boost vaccination regimens. In conclusion, these mutants have shown the potential to provide a model for the study of *M. tuberculosis* as well as a more stable, safe, and effective alternative to the BCG vaccine.

## Figures and Tables

**Table 1:** Auxotrophic strains of *M. bovis* BCG.

Auxotrophy	Complementation Plasmid	Use	Reference
Lysine (*lysA5*)	Episomal ^a^ pJH222.HIVA^CAT^	HIV-TB recombinant vaccine	[11]
Lysine (*lysA5*)	Integrative ^a^ p2auxo.HIVconsv1^int^ and p2auxo.HIVconsv2 ^int^	HIV recombinant vaccine	[17]
Lysine (*lysA5*)	Integrative ^a^ p2auxo.HIVA ^int^	HIV-TB recombinant vaccine	[18]
Lysine (*lysA5*)	Integrative ^a^ p2auxo.HTI ^int^	HIV recombinant vaccine	[19]
Lysine (*lysA5*)	Episomal ^a^ p2auxo.HIVA	HIV–TB recombinant vaccine	[20]
Lysine (*lysA5*)	Episomal ^b^ pJH222	BCG-based HIV vaccine in heterologous prime–boost regimens	[21]
Lysine (*lysA5*)	Episomal ^b^ pJH222 Integrative ^b^ pJH223	molecular characterization of BCG-based HIV vaccines using different expression systems	[22]
Lysine (*lysA5*)	Episomal ^b^ pJH222 Integrative ^b^ pJH223	HIV–TB pediatric vaccine	[23]
Lysine (l*ysA*)	Episomal ^a^ pNL71S1-lysA(kan−)	rBCG vaccine against *Bordetella pertussis*	[12]
Lysine (*lysA5*)	NA	construction of suicide plasmid for Δl*ysA* allelic exchange in slow- and fast-growing mycobacteria	[14]
Leucine (*leuD*)	Episomal ^a^ pUP402 ^#^ and pUP404	construction of a BCG expression system using auxotrophic complementation as a selectable marker	[13]
Leucine *(leuD*)	Episomal ^a,#^ pUP410 + *lipL32*/18kDa and pUP410 + *ligAni*/18kDa	stability of rBCG vaccine against leptospirosis	[24]
Leucine *(leuD*)	Episomal ^a,#^ pUP410::*fbpB* ΔkanR	bovine tuberculosis vaccine based on rBCG overexpressing Ag85B	[25]
Leucine *(leuD*)	Episomal ^a#^ pUP410::85B and pUP410::85BT	stability of rBCG vaccine against bovine tuberculosis	[26]
Leucine *(leuD*)	Episomal ^a,#^ pUP410::*fbpB* ΔkanR	bladder cancer immunotherapy based on rBCG expressing Ag85B	[27]
Leucine (*leuD*)	Episomal ^b^ pMV361	establish the relation between attenuated phenotype of the leucine auxotroph and its ability to grow inside macrophages	[28]
Leucine (*leuCD*)	Episomal ^a^ pSL701,pSL718, pSL720	establishment of quality control parameters for production of anti-HIV, rBCG *ΔleuCD*-based vaccine	[29]
Leucine (*leuD*) Methionine (*sbpA*)	Episomal ^b^ pYUB419 Integrative ^b^ pYUB425	construction and characterization of transposon-induced BCG auxotrophic mutants	[16]
Leucine (*leuD*) Methionine (*sbpA*)	NA	evaluation of protection against tuberculosis without sensitization to the tuberculin skin test	[7]
Methionine (*cysA*)	Episomal ^b^ pSM101EW	screening of a transposon library for characterization of sulphur metabolism	[15]
Pantothenate (*panCD*)	Episomal ^c^ pHS501, pRC501, pHS121, pHS151	combined HIV-TB recombinant vaccine	[30]
Pantothenate (*panCD*)	Episomal ^c^ pHS400	BCG-based HIV vaccine	[31]
Pantothenate (*panCD*)	Episomal ^c^ pHS400	BCG-based HIV vaccine	[32]
Pantothenate (*panCD*)	Episomal ^c^ pTJBCG3	BCG-based HIV vaccine	[33]
Lysine (*lysA5*) Leucine (*leuCD*) Pantothenate (*panCD*) Nicotinamide Adenine Dinucleotide (*nadBC*)	NA	development of a specialized transduction system for generating allelic exchanges in fast- and slow-growing mycobacteria	[34]
Vitamin B6 (*pdx1*)	Integrative ^b^ pMV306	development of rBCG *Δ*ureC::*hlyΔpdx1* for use as a TB vaccine in immunocompromised individuals	[9]
Purines (*purC*)	NA	TB vaccine	[8]

^a^ Auxotrophic complementation provided by plasmid insertion, without employing any antibiotic resistance markers. ^b^ Auxotrophic complementation provided by plasmid insertion; however, antibiotic resistance marker was kept. ^c^ Only antibiotic resistance, but no auxotrophic complementation, provided by plasmid insertion. ^#^ pUP402 and pUP410 have the same plasmid backbone. NA—not applicable; no plasmid was inserted after construction of auxotrophic strain.

## Data Availability

Not applicable.
